# Long-term administration of Western diet induced metabolic syndrome in mice and causes cardiac microvascular dysfunction, cardiomyocyte mitochondrial damage, and cardiac remodeling involving caveolae and caveolin-1 expression

**DOI:** 10.1186/s13062-023-00363-z

**Published:** 2023-03-06

**Authors:** I.-Fan Liu, Tzu-Chieh Lin, Shu-Chi Wang, Chia-Hung Yen, Chia-Yang Li, Hsuan-Fu Kuo, Chong-Chao Hsieh, Chia-Yuan Chang, Chuang-Rung Chang, Yung-Hsiang Chen, Yu-Ru Liu, Tsung-Ying Lee, Chi-Yuan Huang, Chih-Hsin Hsu, Shing-Jong Lin, Po-Len Liu

**Affiliations:** 1 Institute of Clinical Medicine, National Yang Ming Chiao Tung University, Taipei, 112304 Taiwan; 2 Heart Center, Cheng Hsin General Hospital, Taipei, 112401 Taiwan; 3 Graduate Institute of Clinical Medicine, College of Medicine, Kaohsiung Medical University, Kaohsiung, 807378 Taiwan; 4 Division of Cardiology, Department of Internal Medicine, Kaohsiung Medical University, Kaohsiung, 807378 Taiwan; 5 Department of Medical Laboratory Science and Biotechnology, Kaohsiung Medical University, Kaohsiung, 807378 Taiwan; 6 Graduate Institute of Natural Products, College of Pharmacy, Kaohsiung Medical University, Kaohsiung, 807378 Taiwan; 7 Graduate Institute of Medicine, College of Medicine, Kaohsiung Medical University, Kaohsiung, 807378 Taiwan; 8 Department of Internal Medicine, Kaohsiung Municipal Ta-Tung Hospital, Kaohsiung Medical University Hospital, Kaohsiung Medical University, Kaohsiung, 807378 Taiwan; 9 Department of Internal Medicine, School of Medicine, College of Medicine, Kaohsiung Medical University, Kaohsiung, 807378 Taiwan; 10 Division of Cardiovascular Surgery, Department of Surgery, Kaohsiung Medical University Hospital, Kaohsiung Medical University, Kaohsiung, 807378 Taiwan; 11 Department of Surgery, Faculty of Medicine, College of Medicine, Kaohsiung Medical University, Kaohsiung, 807378 Taiwan; 12 Department of Mechanical Engineering, National Cheng Kung University, Tainan, 701401 Taiwan; 13 Department of Medical Science, National Tsing Hua University, Hsinchu, 300044 Taiwan; 14 Institute of Biotechnology, National Tsing Hua University, Hsinchu, 300044 Taiwan; 15 Graduate Institute of Integrated Medicine, College of Chinese Medicine, China Medical University, Taichung, 404333 Taiwan; 16 Department of Psychology, College of Medical and Health Science, Asia University, Taichung, 413305 Taiwan; 17 Department of Respiratory Therapy, College of Medicine, Kaohsiung Medical University, Kaohsiung, 807378 Taiwan; 18 Department of Internal Medicine, College of Medicine, National Cheng Kung University Hospital, National Cheng Kung University, Tainan, 701401 Taiwan; 19 Cardiovascular Research Center, National Yang Ming Chiao Tung University, Taipei, 112304 Taiwan; 20 Department of Medical Research, Taipei Veterans General Hospital, Taipei, 112201 Taiwan; 21 Taipei Heart Institute, Taipei Medical University, Taipei, 110301 Taiwan; 22 Heart Center, Cheng-Hsin General Hospital, Taipei, 112401 Taiwan; 23 Department of Medical Research, Kaohsiung Medical University Hospital, Kaohsiung, 807378 Taiwan; 24 Orthopaedic Research Center, Kaohsiung Medical University, Kaohsiung, 807378 Taiwan

**Keywords:** Metabolic syndrome, Caveolae, Caveolin-1, Endothelial dysfunction, Mitochondrial remodeling, Cardiac mitochondria dysfunction

## Abstract

**Background:**

Long-term consumption of an excessive fat and sucrose diet (Western diet, WD) has been considered a risk factor for metabolic syndrome (MS) and cardiovascular disease. Caveolae and caveolin-1 (CAV-1) proteins are involved in lipid transport and metabolism. However, studies investigating CAV-1 expression, cardiac remodeling, and dysfunction caused by MS, are limited. This study aimed to investigate the correlation between the expression of CAV-1 and abnormal lipid accumulation in the endothelium and myocardium in WD-induced MS, and the occurrence of myocardial microvascular endothelial cell dysfunction, myocardial mitochondrial remodeling, and damage effects on cardiac remodeling and cardiac function.

**Methods:**

We employed a long-term (7 months) WD feeding mouse model to measure the effect of MS on caveolae/vesiculo-vacuolar organelle (VVO) formation, lipid deposition, and endothelial cell dysfunction in cardiac microvascular using a transmission electron microscopy (TEM) assay. CAV-1 and endothelial nitric oxide synthase (eNOS) expression and interaction were evaluated using real-time polymerase chain reaction, Western blot, and immunostaining. Cardiac mitochondrial shape transition and damage, mitochondria-associated endoplasmic reticulum membrane (MAM) disruption, cardiac function change, caspase-mediated apoptosis pathway activation, and cardiac remodeling were examined using TEM, echocardiography, immunohistochemistry, and Western blot assay.

**Results:**

Our study demonstrated that long-term WD feeding caused obesity and MS in mice. In mice, MS increased caveolae and VVO formation in the microvascular system and enhanced CAV-1 and lipid droplet binding affinity. In addition, MS caused a significant decrease in eNOS expression, vascular endothelial cadherin, and β-catenin interactions in cardiac microvascular endothelial cells, accompanied by impaired vascular integrity. MS-induced endothelial dysfunction caused massive lipid accumulation in the cardiomyocytes, leading to MAM disruption, mitochondrial shape transition, and damage. MS promoted brain natriuretic peptide expression and activated the caspase-dependent apoptosis pathway, leading to cardiac dysfunction in mice.

**Conclusion:**

MS resulted in cardiac dysfunction, remodeling by regulating caveolae and CAV-1 expression, and endothelial dysfunction. Lipid accumulation and lipotoxicity caused MAM disruption and mitochondrial remodeling in cardiomyocytes, leading to cardiomyocyte apoptosis and cardiac dysfunction and remodeling.

## Background

Metabolic syndrome (MS) is a set of clinical conditions, such as insulin resistance, hyperglycemia, systemic arterial hypertension (SAH), dyslipidemia, obesity, and a large abdominal circumference [1]. MS alterations cause multiple clinical manifestations and are recognized as leading risk factors for cardiovascular diseases. Long-term (15 weeks) feeding with a Western diet (WD) (45% kcal fat) causes MS, increased heart mass, and exhibited critical features of pathological hypertrophy, including fibrosis and upregulation of pathological hypertrophy-associated genes [2]. In addition, MS can result in cardiac injury, including diastolic dysfunction, impaired calcium handling [3], diabetic cardiomyopathy [4], and heart failure [5]. Lipid droplets (LD) regulate intracellular lipid storage and lipid metabolism of neutral lipids such as cholesteryl esters, and triglycerides such as triolein. Hyperlipidemia causes lipid overload in adipocytes and various organs, especially the eyes, kidneys, liver, blood vessels, and heart, and accumulation leads to lipotoxicity [6]. There are two modes of macromolecular (such as cholesterol and fatty acid) extravasation transport across the endothelium: the vesiculo-vacuolar organelle (VVO) and caveolae. VVO is one of the pathways for macromolecule extravasation in endothelial cells of the micro-circulation and tumors microvasculature [7]. Through all endothelial cells, VVO forms a conduit from the luminal surface of the vascular endothelial cells to the other side of the endothelial cells near the surface of the tissue end [8]. The significant increase in tumor vascular permeability signature may be attributed to the upregulation of VVO function [7]. However, further research is required to determine whether hyperlipidemia or MS affects the generation of VVO in myocardial microvascular endothelial cells.

Previous studies have demonstrated that diabetes modulates changes in the cardiac sarcolemma, including alterations reflecting lipid metabolism, lipid transport modification, caveolae remodeling and regulation of caveolins (CAVs) protein expression, prominent structures of T-tubules and gap junctions, and functional remodeling [4]. Caveolae are 50‒100 nm cell-surface plasma membrane invagination and expression in various cells, including fibroblasts, smooth muscle cells, epithelial cells, adipocytes, endothelial cells, and cardiomyocytes [9]. Caveolae are highly expressed and abundant in vascular endothelial cells to regulate endothelial vesicular trafficking, signal transduction, cholesterol transportation, cell-to-cell communication, and endothelial function [10, 11]. CAVs, cholesterol-binding oligomeric proteins, regulates intracellular cholesterol transport by a complex process involving the caveolae, mitochondria, endoplasmic reticulum (ER), vesicles, and Golgi network [12]. CAVs (CAV-1, -2, and -3) are protein families controlling the biogenesis and function of caveolae, which are plasma membrane omega-like invaginations that serve as the primary site of critical cellular processes. CAV-1 and CAV-2 are ubiquitously expressed in all cell types, whereas CAV-3 is mainly found in cardiomyocyte and skeletal muscles. CAV-1 is an oncogenic membrane protein associated with endocytosis, extracellular matrix organization, cholesterol distribution, lipid disorders, cell migration, and signaling [13]. In addition, CAV-1 modulates cell metabolism, including glycolysis, mitochondrial bioenergetics, glutaminolysis, fatty acid metabolism, mitophagy, and autophagy [14, 15]. However, further research is necessary to determine whether heart related diseases caused by MS and cardiac remodeling will directly affect the expression of caveolae in cardiac vascular endothelium and cardiomyocytes.

MS has been linked to oxidative stress production, endothelial cell activation, endothelial cell dysfunction, and the pathogenesis of macrovascular diseases [16]. The normal endothelial function includes dynamic maintenance of vascular tone, vascular permeability, angiogenesis, and antioxidant, anti-inflammatory, and antithrombotic reactions. Vascular endothelial dysfunction is a complex pathological process including impaired endothelium-dependent vasodilation, increased vascular permeability, altered expression of adhesion factors between endothelium, increased oxidative stress, increased expression of inflammatory hormones, and increased leukocyte adhesion. Increased adherence, altered endothelial cell metabolism, endothelial cell senescence, endothelial-mesenchymal transition, and endothelial damage or death are common causes of endothelial dysfunction. [17, 18]. In addition, the progression of metabolic and cardiovascular diseases caused by a Western diet is associated with endothelial activation and insulin resistance [19]. Furthermore, a recent study indicated that diabetes mellitus and cardiometabolic disease may have clinically significant adverse effects on microvascular function [20]. However, the effect and regulation of MS on cardiac microvascular endothelial cells remains unclear.

Mitochondrial dynamics include fission, fusion, shape transition (MST), and mitochondrial transport through the cytoskeleton [21]. However, the effect of MS-induced lipotoxicity on the expression of cardiac mitochondrial-CAV-1 and MST needs to be further clarified. The mitochondria-associated endoplasmic reticulum membrane (MAM) is a cellular structure formed by the proximity of two organelles: the mitochondria and ER [22]. MAM is involved in fundamental biological processes including ER stress, lipid and calcium (Ca^2+^) homeostasis, mitochondrial dynamics, and other related cellular reactions, such as autophagy, mitochondrial autophagy, inflammation, and apoptosis [23]. MAM dysfunction is directly related to the pathological progression of ischemia–reperfusion, diabetic cardiomyopathy, heart failure, pulmonary hypertension, and systemic vascular disease [23, 24]. In addition, MAMs play a crucial role in regulating physiology, pathophysiology, and cellular metabolism. MAM dysfunction is associated with MS, including the downregulation of insulin signaling and accelerated progression of hyperlipidemia, obesity, and hypertension [22]. However, the effects of MS-induced cardiac incapacitation and remodeling on intramyocardial MAM and myocardial mitochondrial damage need further investigation. This study aimed to investigate the correlation between CAV-1 expression and abnormal lipid accumulation in the endothelium and myocardium in WD-induced MS, and the occurrence of myocardial microvascular endothelial cell dysfunction, myocardial mitochondrial remodeling, and damage effects on cardiac remodeling and function.

## Results

### Long-term Western diet feeding caused obesity and MS in vivo

**Fig. 1:**
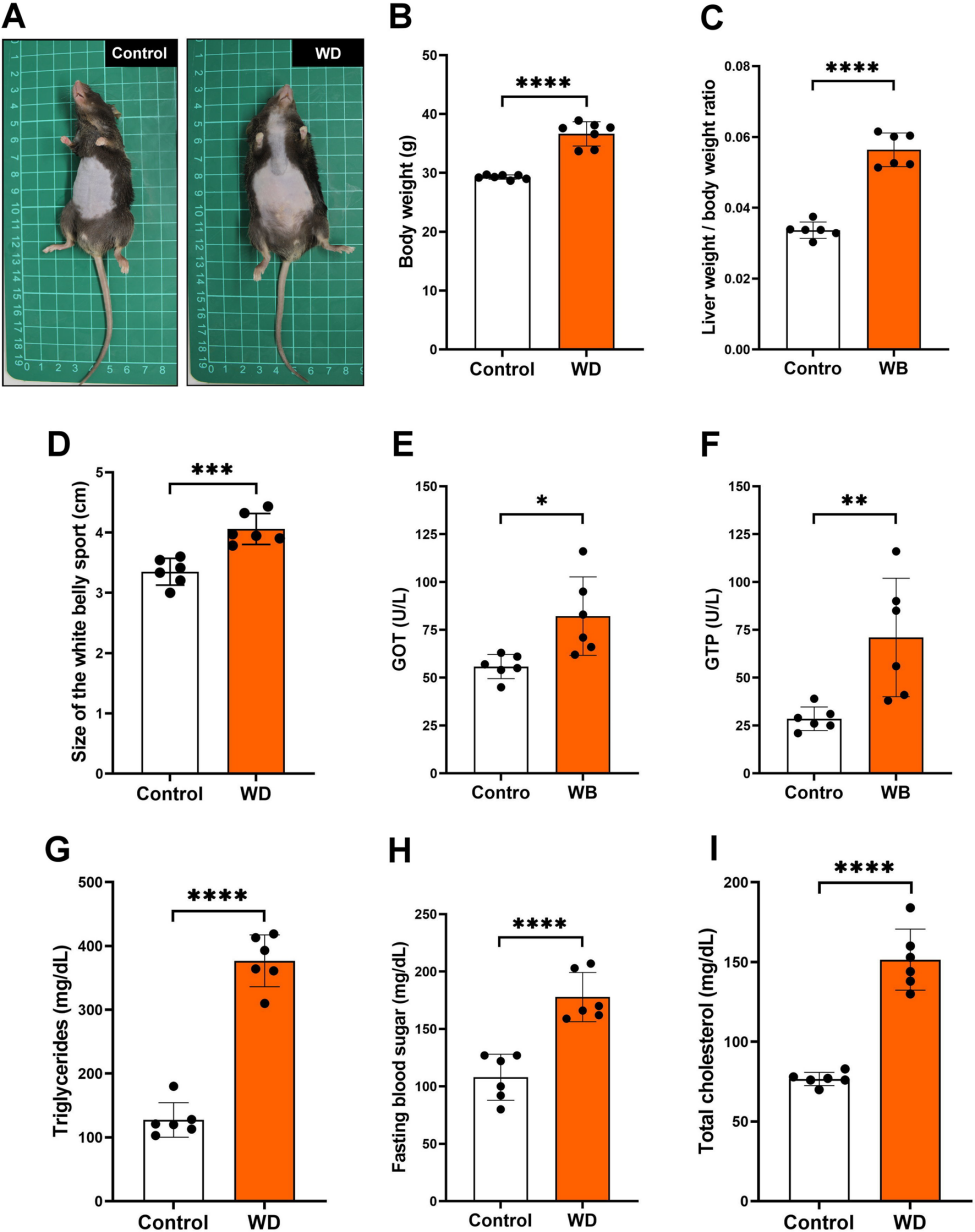
Long-term Western diet feeding caused obesity and MS in aged mice. Mice were fed either the normal feed diet (Control) or Western diet (WD) for 7 months. **A** Representative photo of mice after seven months of control and WD groups. **B** Body weight, **C** liver weight/body weight ratio, **D** size of the white belly sport, **E** GOT, and **F** GTP, **G** triglycerides, **H** fasting blood sugar, and **I** total cholesterol change after seven months of control or WD feeding. *P < 0.05; **P < 0.01; ***P < 0.001; ****P < 0.0001

To determine whether the long-term Western diet induced obesity and MS in aging mice, we compared the results of the control group (animals fed with a regular diet) and the Western diet group after 7 months. Our data showed that a long-term Western diet caused obesity significantly (Fig. 1A) and increased mice body weight (Fig. 1B), liver/body weight ratio (Fig. 1C), and abdominal circumference (Fig. 1D). Furthermore, we examined the biochemical values related to MS, including glutamic oxaloacetic transaminase (GOT) (Fig. 1E), guanosine triphosphate (GTP) (Fig. 1F), triglycerides (Fig. 1G), fasting blood sugar (Fig. 1H), and total cholesterol (Fig. 1I) compared with the control group. Our data indicated that the long-term Western diet caused MS in vivo.

### The caveolae and VVO formation and CAV-1 expression in cardiovascular tissue in vivo

**Fig. 2:**
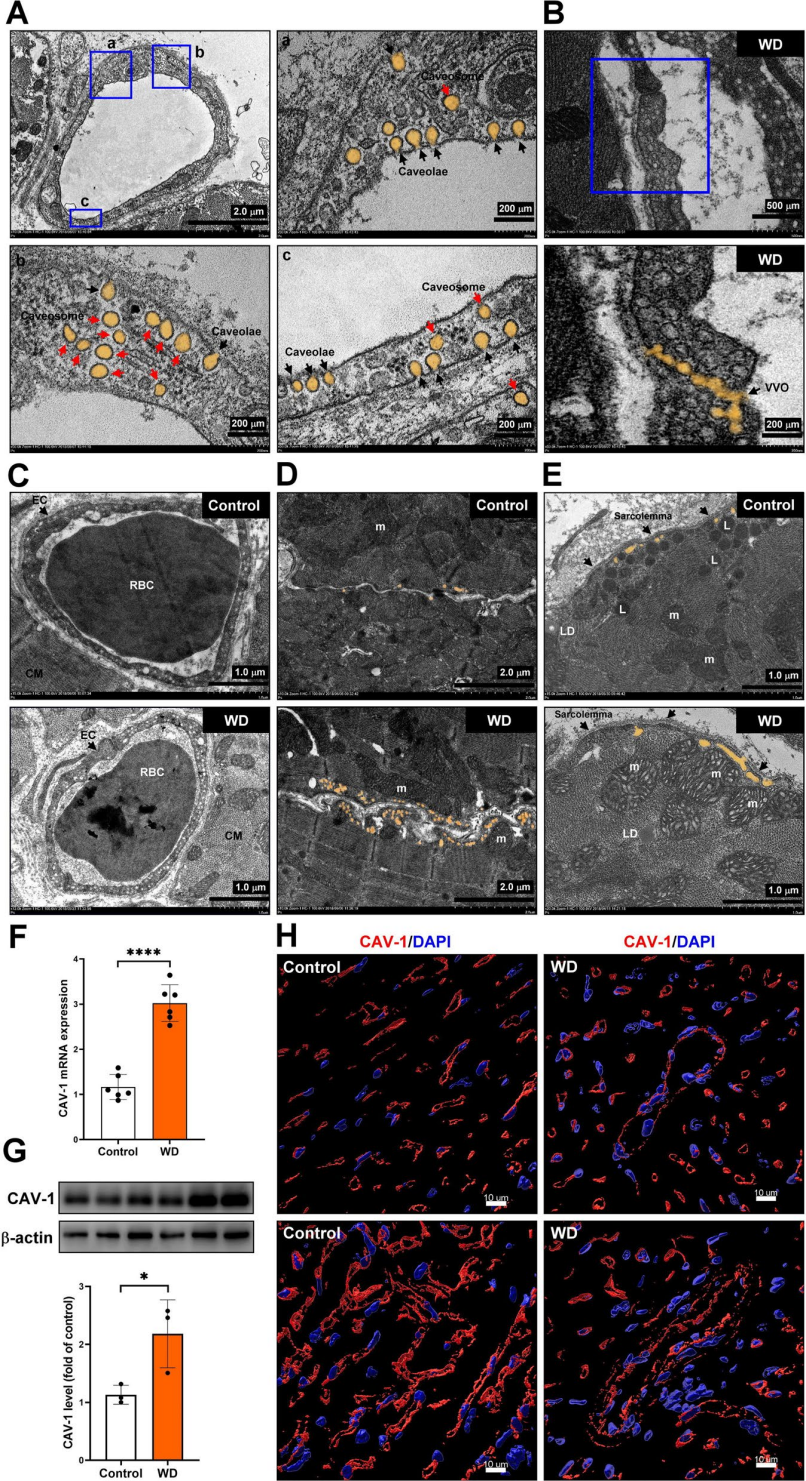
The caveolae and VVO formation and CAV-1 expression in cardiovascular tissue in vivo. **A**,** B** The caveolae, and caveosome expression in cardiac vascular endothelial cells were examined by TEM assay. **C** The VVO formation only in the WD group was analyzed by TEM assay. **D**, **E** The expression of caveolae in the junction between perivascular cardiomyocytes and cardiomyocytes was analyzed by TEM assay. **F**–**H** The CAV-1 mRNA and protein expression were measured by real-time PCR, Western blot and immunostaining. *P < 0.05; ****P < 0.0001

The TEM assay was used to explore the caveolae expression in cardiac microvascular endothelial cells. Our results showed the caveolae and caveosome expression on the surface of the endothelial and intracellular (caveosome) membranes (Fig. 2A). Furthermore, the VVO formation was only found in the microvascular endothelial cells of the Western diet-induced MS group (Fig. 2B). The TEM assay was used to identify MS regulation in caveolae expression in microvascular endothelial cells (Fig. 2C), the junction between perivascular cardiomyocytes (Fig. 2D) and subsarcolemma cardiomyocytes (Fig. 2E). Our results showed that the Western diet-induced MS significantly increased caveolae and caveosome expression in endothelial cells. In addition, the Western diet-induced MS promoted mitochondrial and caveolae adhesion in subsarcolemmal cardiomyocytes; however, in the control group, more lysosomes aggregated in the subsarcolemmal region. It has been reported that the expression of caveolin is upregulated in diabetic myocardium [25]. The real-time polymerase chain reaction (PCR), Western blot, and immunostaining assay were used to examine the expression of CAV-1 in cardiac tissue (Fig. 2F–H). Our data showed that Western diet-induced MS significantly increased CAV-1 mRNA and protein expression in cardiac tissue.

### The MS-induced cardiac microvascular endothelial dysfunction in vivo

**Fig. 3:**
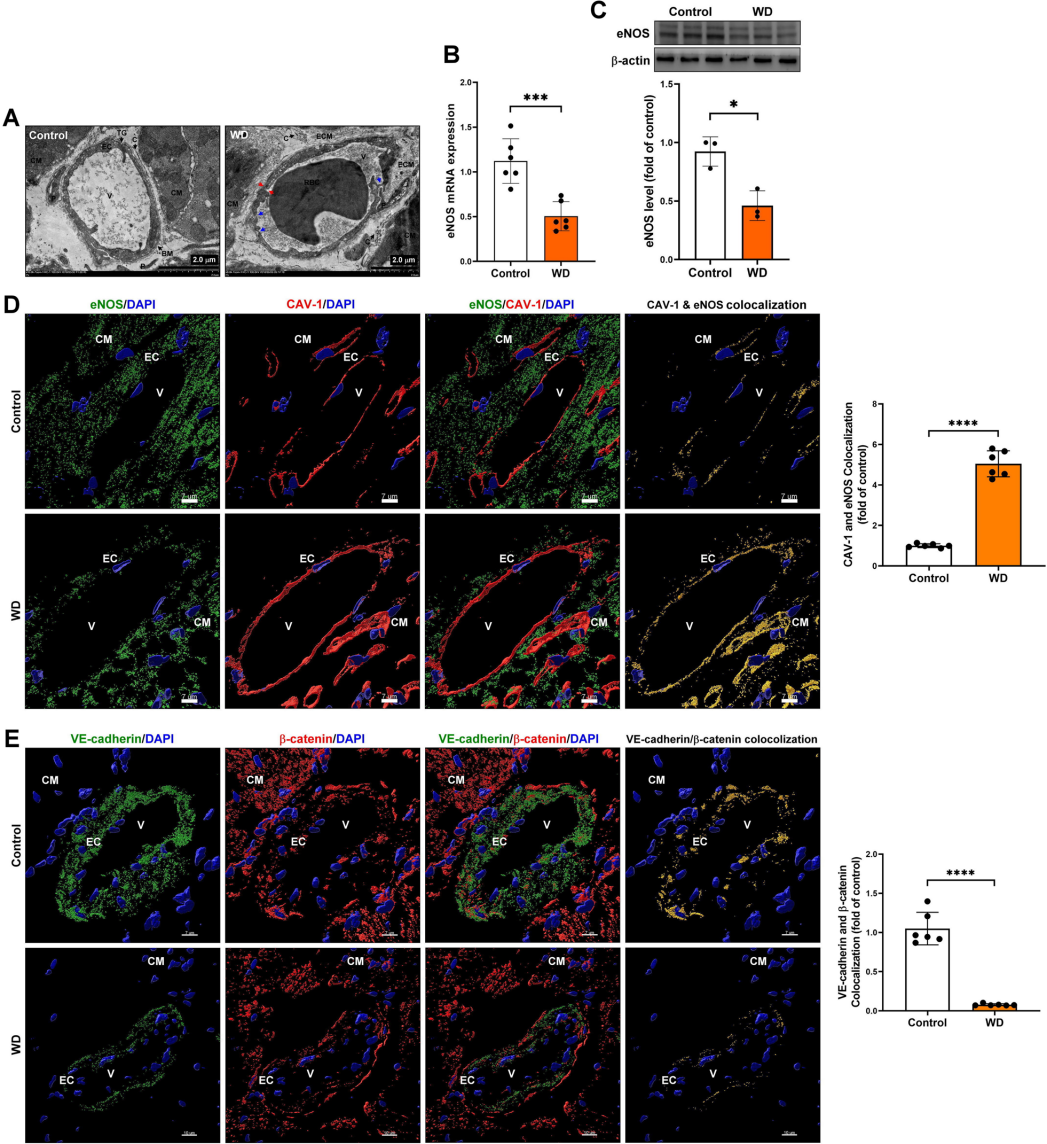
The MS induced cardiac microvascular endothelial dysfunction in vivo. **A** The cardiac microvascular ultrastructure change was examined by TEM assay. EC, endothelial cell; CM, cardiomyocyte; V, vascular; BM, basement membrane; ECM, extracellular matrix; P, pericyte cells; red arrow, endothelial cell rupture; blue arrow, damaged endothelial cell. **B, C** The eNOS mRNA and protein expression were examined by real-time PCR and Western blot assay. **D** The caveolin-1 and eNOS expression and interaction was measured by immunostaining and confocal microscope assay. **E** Endothelial adhesion molecular VE-cadherin and β-catenin expression and interaction was measured by immunostaining and confocal microscope assay. *P < 0.05; ***P < 0.001; ***P < 0.001

Endothelial dysfunction is a pathophysiological event involving endothelial cell activation and endothelial dysfunction. Endothelial cell activation is the first significant change, including pro-inflammatory and pro-thrombotic endothelial cell activation, resulting in decreased eNOS expression, and reduced nitric oxide (NO) bioavailability [26]. The cardiac microvascular ultrastructure change was examined using TEM assay. Our data showed that in addition to exhibiting more caveolae, microvascular endothelial cells in the Western diet-induced MS group had impaired basement membrane integrity, increased extracellular matrix collagen fiber accumulation, decreased pericyte cell number, destabilization of interendothelial cell junctions, and increased endothelial cells bleb, damage, or rupture (Fig. 3A). The real-time PCR, Western blot, and immunostaining assay were used to examine the level of eNOS in cardiac tissue (Fig. 3B–C). Our data showed that Western diet-induced MS significantly decreased eNOS mRNA and protein expression in cardiac tissues. Previous studies have demonstrated that in the endothelium, NO is synthesized by eNOS and is negatively regulated by Cav-1; dysregulation of eNOS and CAV-1 contribute to endothelial dysfunction in diabetic dyslipidemia [27, 28]. The immunostaining and confocal microscope assays were used to analyze eNOS and CAV-1 interaction. Our results demonstrated that MS significantly increased eNOS and CAV-1 interaction in microvascular endothelial cells and cardiomyocytes (Fig. 3D). In addition, the immunostaining and confocal microscope assays were used to examine vascular endothelial cadherin (VE-cadherin) and β-catenin expression and interaction to explore the endothelial adhesion molecular change in MS. Our results showed that MS reduced VE-cadherin expression levels and downregulated VE-cadherin and β-catenin interaction (Fig. 3E).

### Lipid accumulation in cardiovascular tissue regulated by CAV-1 in MS mice

**Fig. 4:**
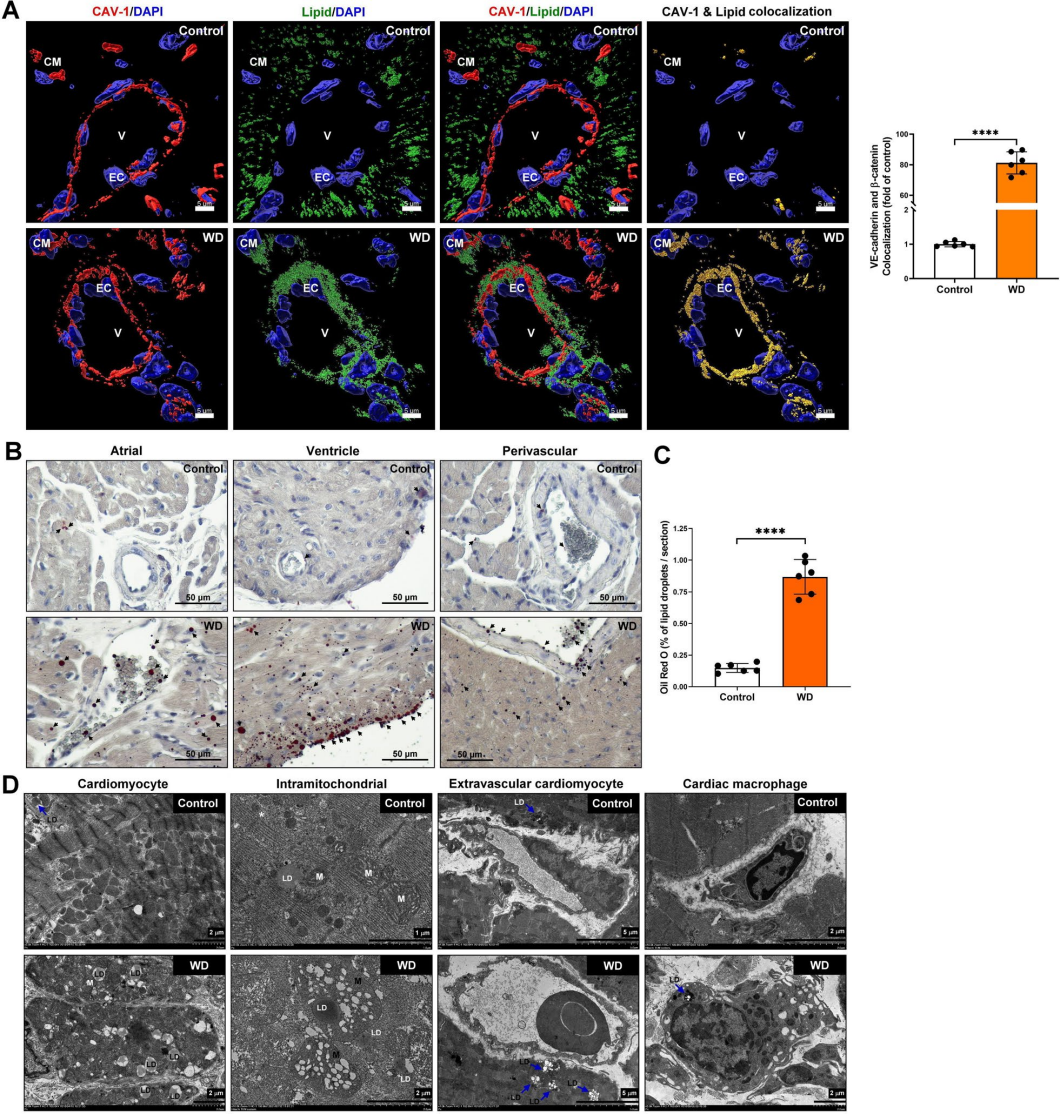
Lipid accumulation in cardiovascular tissue regulated by CAV-1 in vivo. **A** The immunostaining was used to identify lipid droplet transport from the cardiovascular to the myocardium mediated by endothelial cell`s CAV-1. Red color, CAV-1; green color, lipid, blue color DAPI, yellow color CAV-1/lipid colocalization. EC, endothelial cell; CM, cardiomyocyte; V, vascular. **B**, **C** The oil-Red O staining was used to confirm and quantify of lipid droplets accumulation in cardiac tissue. Data are presented as the mean ± SEM; ****P < 0.0001. **D** The lipid droplets accumulation in cardiomyocyte, intramitochondrial, extravascular cardiomyocyte and cardiac macrophages were examined by TEM assay. LD, lipid droplet; L, lysosome

CAV-1 plays a crucial role in caveolae-mediated low-density lipoprotein (LDL) uptake and cholesterol transcytosis across the endothelium [29]. In MS patients presenting with clinical symptoms such as systemic arterial hypertension, dyslipidemia, obesity, and increased abdominal circumference, there is a marked upregulation of CAV-1 mRNA expression [1]. The immunostaining and confocal microscope were used to examine the regulation of CAV-1 in lipid transport in vascular endothelial cells. Our data showed that the CAV-1 and lipid colocalization in cardiovascular endothelial cells was significantly increased in the Western diet-induced MS group (Fig. 4A). In addition, to confirm the lipid accumulation in cardiac endothelial cells and atrial/ventricular cardiomyocytes, Oil-red O staining was performed. Our data indicated that the lipid drop was significantly higher in the Western diet-induced MS group (Fig. 4B–C). Furthermore, the lipid distribution in cardiac tissue was examined using TEM assay. Our data indicated that the Western diet-induced MS increased the lipid accumulated in cardiomyocytes, intramitochondrial, extravascular cardiomyocytes, and cardiac macrophages (Fig. 4D). These results indicated that Western diet-induced MS increased CAV-1 binding to lipids in vascular endothelial cells, further regulating lipid transport from blood vessels to cardiomyocytes, mitochondria, and macrophages for accumulation.

### Disruption of MAM formation and mitochondrial remodeling in MS mice cardiomyocytes

**Fig. 5:**
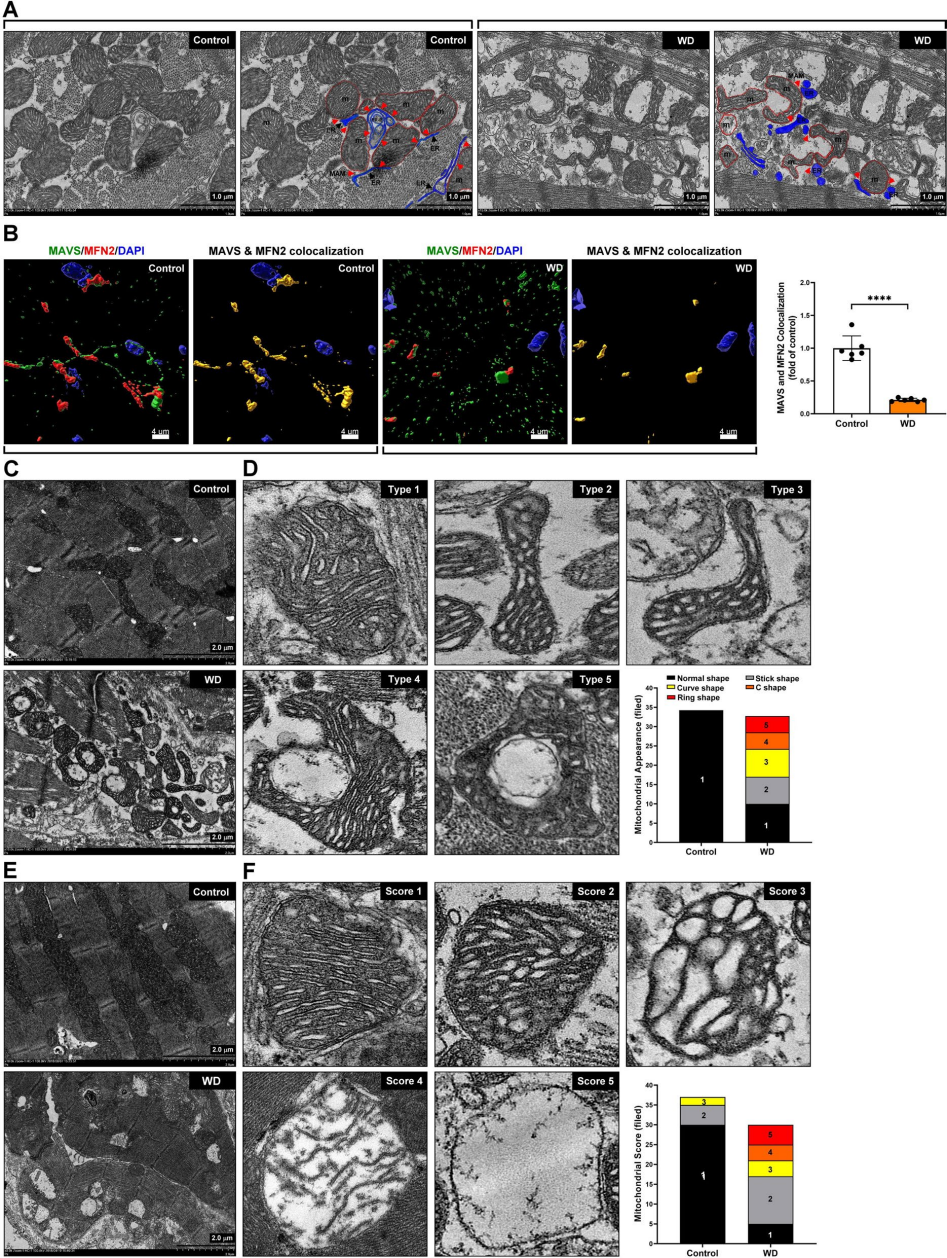
Disruption of MAM formation and mitochondrial remodeling in MS mice cardiomyocytes. **A** The mitochondria-associated endoplasmic reticulum membrane (MAM) was examined using TEM assay. ER, blue color, endoplasmic reticulum; m, red, color, mitochondria; ER-P, reticulophagy; MAM, red arrow. **B** MAM proteins mitochondrial antiviral signaling (MAVS) and mitofusin 2 (MFN2) expression and colocalization were examined by immunostaining and confocal microscopy. **C**, **D** The mitochondrial morphological remodeling was measured and quantified using TEM analysis. **E**, **F** Mitochondrial distribution and damage score quantification by TEM analysis

The MAM is a cellular structure that connects and mediates communication between the ER and mitochondria. MAM is involved in calcium signaling, lipid metabolism, oxidative stress generation in mitochondria and ER, protein folding, mitochondrial dynamics, and autophagy. In addition, MAMs play a crucial role in cellular metabolism, and the dysfunction of MAMs is directly related to neurodegenerative diseases [30], heart failure [24], and MS [22]. TEM assay was performed to further confirm that MAM changes in MS`s cardiomyocytes. Our experimental results found that the remodeling and deformation of mitochondria and ER significantly reduced the area of MAM in the cardiomyocytes of mice with MS (Fig. 5A). Moreover, to further confirm ER-mitochondria dis-communication, immunostaining and confocal microscope were used to examine the MAM maker proteins, MAVS and MFN2, expression and colocalization. Our results showed that MAVS and MFN2 protein expression significantly reduced and lacked interaction in the myocardium of mice with MS compared to the control group (Fig. 5B). These results indicate that the ER-mitochondria communication was disrupted in the Western diet-induced MS group.

An increase in plasma cholesterol and TG has been associated with lipid toxicity and the development of mitochondrial dysfunction [31]. The TEM assay was used to examine the damaged mitochondrial ultrastructure and modulated dynamic caused by the Western diet. Our data indicated that the mitochondrial ultrastructure was changed, including the formation of mitochondrial fission, increased mitochondrial swelling, and accumulation of vacuolized mitochondria (Fig. 5C). It is worth mentioning that in the Western diet-induced MS group, mitochondria surround damaged organelles (vaccinated mitochondria or damaged ER) through self-remodeling. This process resulted in changes in mitochondrial morphology and structure, such as rod-shaped, curved, and ring-shaped mitochondria; however, this phenomenon was rare in the control group (Fig. 5D). We further evaluated the mitochondrial damage by establishing a mitochondrial damage assessment score. The morphological appearance of mitochondrial damage includes score 1: compact and regular cristae, dense mitochondrial matrix; score 2: cristae gape increasing and irregular, reducing mitochondrial matrix density, slight swelling; score 3: cristae irregular arrangement, mitochondrial matrix transparentizing, moderate swelling; score 4: cristae fragment, mitochondrial inner membrane dissociated with cristae, sever swelling; and score 5: the disappearance of mitochondrial contents, mitochondria vacuolization (Fig. 5E–F). The distribution of mitochondria in mature cardiomyocytes presents three forms, which are unique compared to other myocardial mitochondria.

The three types are as follows: fibrillar mitochondria, which are densely distributed in myofibril and parallel to the sarcomere; subsarcolemmal mitochondria, which are distributed irregularly under the sarcolemma; paranuclear mitochondria, which are distributed around the nucleus [32]. Diabetes and cardiovascular disease are thus closely related to an imbalance between mitochondrial fission and fusion [33]. Our results demonstrated that dynamic mitochondrial morphology (fusion and fission) differs in control and WD groups. In the control group, the long spheroid and fusion-type mitochondria are densely confined among myofibrils and adjacent to the sarcomere. Moreover, compared with the control group, mitochondria in WD become fission-type, smaller, have large vacuoles and damage under electron microscopy (Fig. 5E). The results showed that Western diet-induced MS caused dynamic mitochondrial morphology change and damage.

### The pathological cardiac remodeling in MS mice

**Fig. 6:**
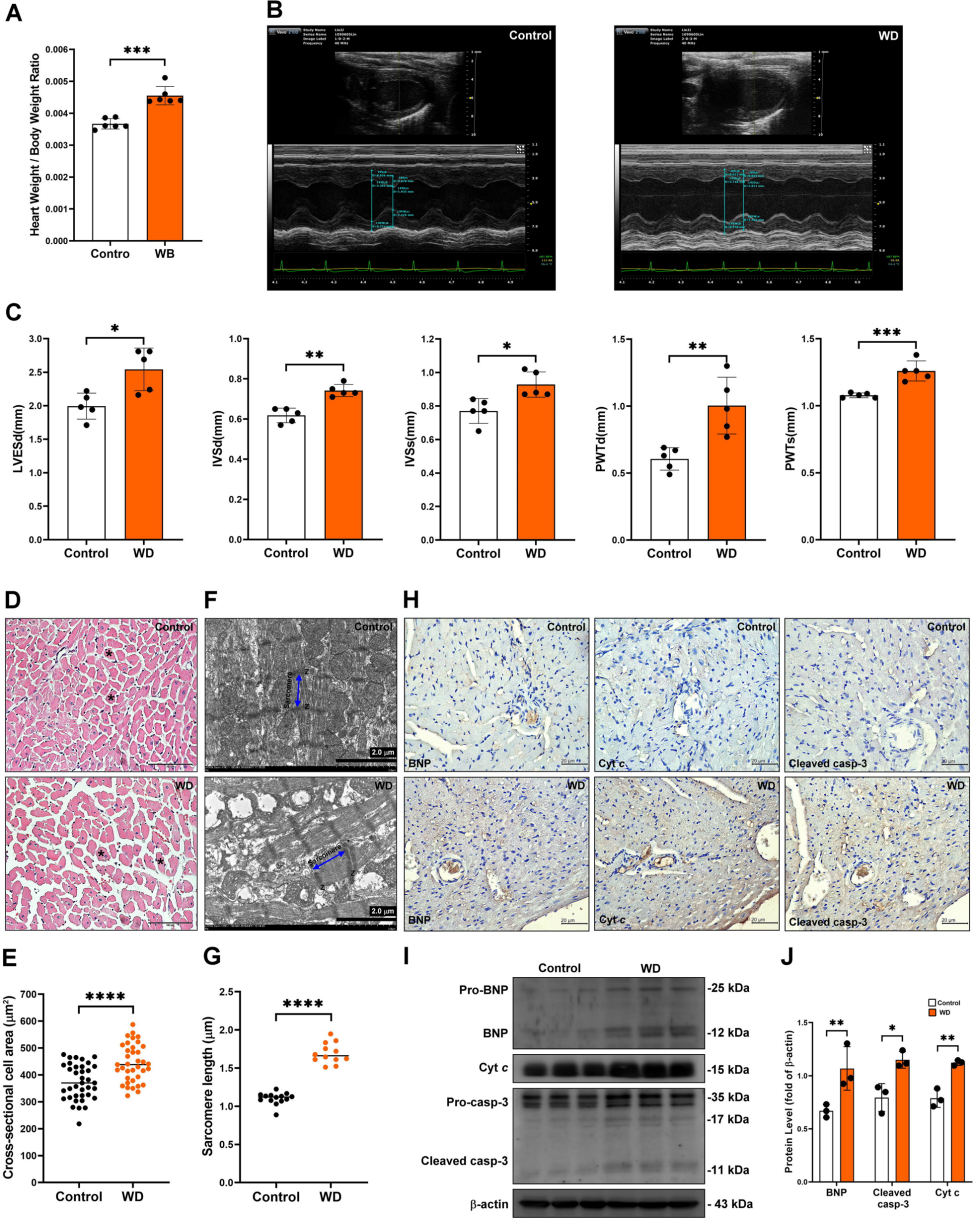
The pathological cardiac remodeling in the Western diet-induced MS mice. **A** Heart weight/body weight ratio was measured in vivo. **B**, **C** Cardiac function was examined by M‐mode echocardiography left ventricular end-systolic diameter (LVESd), inter-ventricular septal thickness in diastole (IVSd), inter-ventricular septal thickness in systole (IVSs), posterior LV wall thickness in diastole (PWTd), and posterior LV wall thickness in systole (PWTs). **P < 0.01, ***P < 0.001. **D**, **E** Cross-section cell area was examined and quantified by H&E staining. ***P < 0.0001. **F**, **G** Sarcomere length was measure and quantified using TEM analysis. ***P < 0.0001. **H**–**J** The BNP, cytochrome *c* (Cyt *c*), and cleaved caspase-3 (cleaved casp-3) protein expression in cardiac tissue were examined and quantified by immunohistochemistry and western blot assay. *P < 0.05, **P < 0.01

Heart weight/body weight ratio was significantly increased in the long-term Western diet-feeding group (Fig. 6A). Therefore, we explored the cardiac function by M-mode echocardiography. Our results indicated that long-term Western diet feeding increased left ventricular internal diameter in diastole (LVIDd) and decreased stock volume (SV), ejection fraction (EF), and fractional shortening (FS). These results indicated that the long-term Western diet feeding caused cardiac remodeling (Fig. 6B). In contrast, long-term Western diet-induced MS induced characteristic myocardial disarray, myofiber thickening, and significantly increased extracellular matrix area in the control group as observed using hematoxylin and eosin (H&E) staining. (Fig. 6C–D). In addition, the sarcomere length and myofibril fragment were increased in the long-term Western diet feeding group. As a result, the worst myofibrillar structure was observed, the overlapping structure of thick and thin filaments was destroyed, muscle structure was severely broken, and the Z-disk distorted and weakened (Fig. 6E–F). Furthermore, the long-term Western diet feeding increased the expression of myocardial damage marker B-type natriuretic peptide (BNP), cytochrome *c*, and cleavage caspase-3 protein (Fig. 6G–I). Collectively, these data indicated that the long-term Western diet feeding significantly caused cardiomyocyte hypertrophy, decreased the overall integrity of the myofibrils, and caused myocardial damage.

## Discussion

Lipotoxicity is the accumulation of lipid intermediates and final products in non-adipose tissue, including the vascular endothelial cells, kidney, skeletal muscle, and heart, leading to mitochondrial dysfunction and increased endoplasmic reticulum stress, accelerating inflammation and cellular dysfunction [34]. Lipotoxicity is essential in the progression of MS, atherosclerosis, diabetes, and heart failure [35]). Previous studies have shown that dyslipidemia can affect the regulation of nitric oxide (NO) signaling in endothelial cells. In addition, dyslipidemia can promote excessive production of ROS in endothelial cells and cause mitochondrial dysfunction in vascular endothelial cells, promoting apoptosis or endothelial dysfunction [36]. Therefore, dyslipidemia causes very low-density lipoprotein (VLDL) and LDL to be susceptible to oxidation by macrophages, endothelial cells, and smooth muscle cells to produce cytotoxicity, which further damages vascular endothelial cells or cardiomyocytes and mediates the progression of cardiovascular disease [37, 38]. Our results indicated that Western diet-induced MS caused massive lipid accumulation in endothelial cells, cardiomyocytes, macrophages, and cardiac mitochondria. Furthermore, MS caused lipotoxicity-induced cardiotoxicity, resulting in the progression of heart failure, including abnormal cardiac function (increasing LVESd, IVSd, IVSs, PWTd, and PWTs), high expression of BNP, increased cardiac muscle fiber area and heart weight, and activated the mitochondria-regulated apoptotic pathway to promote cardiomyocyte apoptosis.

Caveolae are specialized lipid rafts on the plasma membrane, including endothelial cells, smooth muscle cells, epithelial cells, fibroblasts, adipocytes, and cardiomyocytes. CAV-1 regulates intracellular lipid transport by binding with free cholesterol, glycolipids, and fatty acids, in the ER, Golgi, mitochondria, and caveolae. Previous studies have demonstrated that CAV-1 regulates the permeability of vascular interendothelial junctions and the expression of VE-Cadherin and β-catenin, and mediates vascular endothelial permeability and endothelial barrier dysfunction [39, 40]. Our results showed that Western diet-induced MS significantly increased the expression of caveolae and CAV-1 in the vascular endothelial cells, enhanced CAV-1 and eNOS interaction, increased the CAV-1 and lipid droplets binding efficiency, and reduced endothelial adhesion molecular expression. We speculate that this may increase the permeability of the vascular endothelium, which promotes lipid deposition to cardiomyocytes and causes lipotoxicity. Previous reports have shown that CAV-1 deficiency promotes autophagy activation in the vascular endothelium, which reduces endothelial inflammation and atherosclerosis progression [41]. However, we did not have CAV-1 knockout mice to determine endothelial/cardiac dysfunction mediated by CAV-1 in the Western diet model. Therefore, this is a limitation of the study.

Relevant studies have confirmed that CAV-1 plays an integral role in maintaining mitochondrial shape, plasticity, and function, and loss of CAV-1 promotes mitochondrial senescence and dysfunction [42–44]. Related studies have indicated that CAV-1 in the ER regulates lipid metabolism, ER stress, and maintenance of MAM function. In addition, CAV-1 deletion in the ER causes apoptosis by ER stress [45]. In our study, cardiomyocytes promoted the increased expression of CAV-1 in response to lipotoxicity and oxidative stress derived from excessive lipid metabolism in MS. However, the overexpression of CAV-1 in the myocardium seemed insufficient to provide mitochondria and endoplasmic reticulum with an anti-stress effect. We speculate that Western diet-induced MS causes disruption of MAM formation and creates an imbalance between mitochondrial and ER stress, promoting mitochondrial dysfunction and myocardial cell damage or death. The stress disorder between mitochondria and ER caused by MS has not been further investigated, which is a limitation to the study.

To maintain the balance of essential functions and dynamics of cells, mitochondria are remodeled by changing their structure and shape to meet intracellular and extracellular demands and stress to the intracellular environment. The dysregulation of mitochondrial remodeling plays a critical role in the progression of many diseases [46]. Changes in the environment dynamics affects cardiac mitochondria between cardiomyocytes and change in size, number, and shape of mitochondria to maintain function. This process, called mitochondrial shape transition (MST), is not associated with mitochondrial fission/fusion. Common MST-regulated mitochondrial shapes include elongated, rounded, and ring mitochondria [21, 47]. MST is mediated by cytoskeleton and the ER [48]. Compared with other types of cells, the morphological changes of mitochondria in cardiomyocytes are significantly less. Our results showed that Western diet-induced MS regulated MST, including stick, curve, C, and ring shape mitochondria. A previous study indicated that mitochondria with hollow chamber structures might be formed due to intrinsic or environmental factors, including aging, accumulation of toxins, drug action, and oxidative stress injury, leading to the formation of hollow chambers with similar inner and outer mitochondrial membrane structures [49]. In addition, these central void mitochondria have otherwise normal cristae and their overall respiratory activity may also be regular [50]. Furthermore, high-fat diet-induced hyperglycemia and hyperlipidemia promote the production of MST, which is directly related to the generation of intracellular ROS. A high-fat diet promotes MST, which significantly increases the elongated shape of mitochondria, and this remodeling process effectively reduces the occurrence of mitochondrial dysregulation, suggesting that mitochondrial remodeling plays an essential role in the challenge of hyperglycemia and dyslipidemia [51].

## Conclusions

**Fig. 7:**
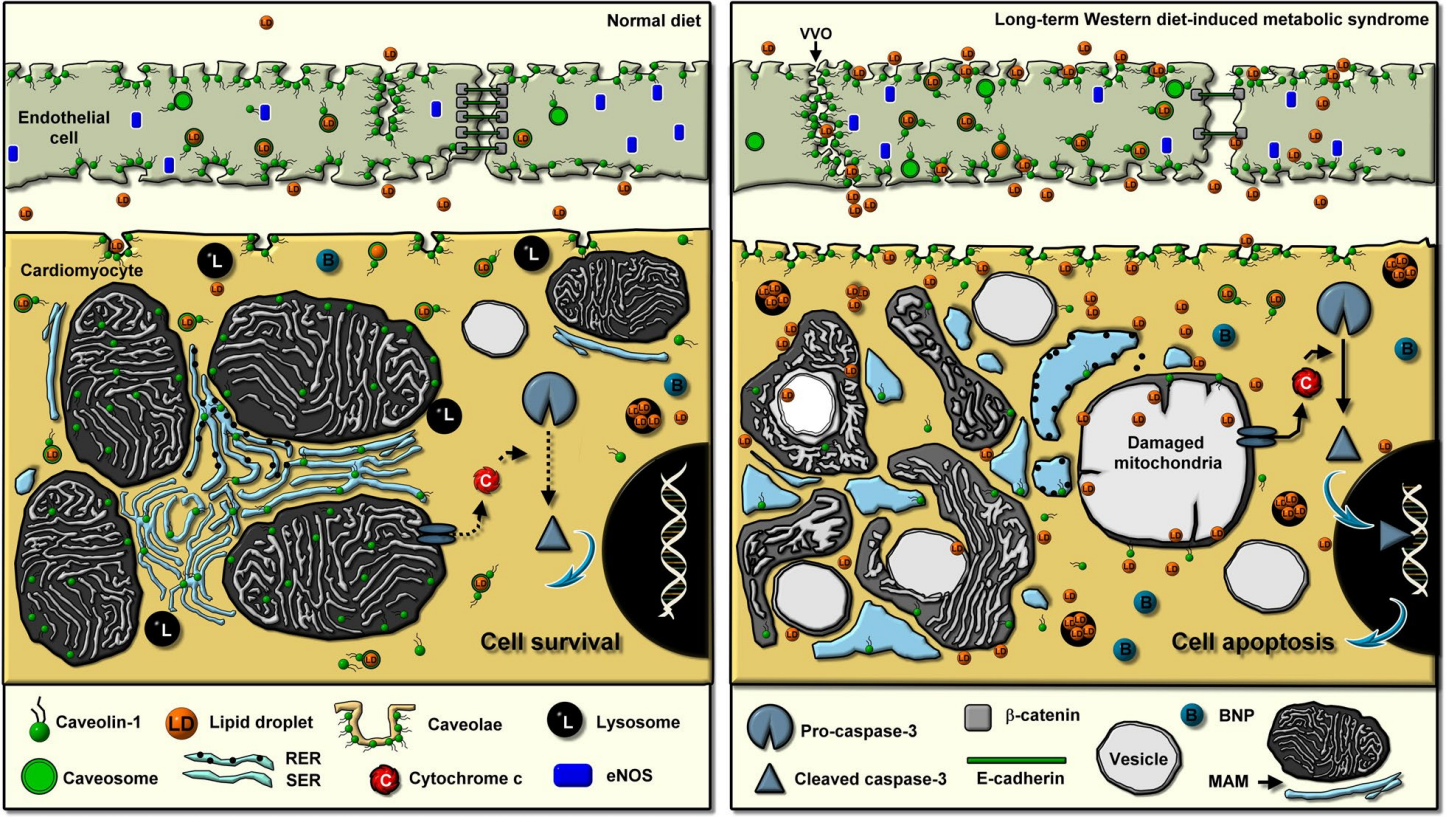
Schematic representation in long-term Western diet-induced MS. Schematic representation of the association between cardiac microvascular dysfunction, caveolae and CAV1 expression, myocyte mitochondrial shape transition and remodeling, and cardiac remodeling in long-term Western diet-induced MS

The Western diet-induced MS regulated cardiac remodeling by increasing caveolae and CAV-1 expression in endothelial cells and myocytes, promoting endothelial dysfunction, increasing lipid accumulation and lipotoxicity in non-adipocyte tissue, disrupting MAM, regulating MST, activating mitochondrial-regulated apoptosis, and resulting in cardiac remodeling (Fig. 7). The protective effect of CAV-1 on the heart can provide a reference for the future clinical treatment of MS-derived heart disease.

## Methods

### Ethical statement

The animal experimental protocol was approved by the Ethics Review Committee for Animal Experimentation of the Kaohsiung Medical University (approval number: 110065).

### Animal model

The administration of the Western diet was modified based on a previous study [52]. Briefly, male C57BL/6J mice (9 weeks old) were purchased from the National Lab Animal Center (Taipei, Taiwan) and housed under standard conditions in an Association for Assessment and Accreditation of Laboratory Animal Care International (AAALAC)-accredited facility (animal center at Kaohsiung Medical University). Mice were fed a normal chow diet and tap water (control group) or Western diet containing 21.2% fat, 48.5% carbohydrate, 17.3% protein by weight (Envigo Teklad Custom Diet, TD.120528, Wisconsin, USA), a high sugar solution (23.1 g/L d-fructose (Sigma-Aldrich, G8270, St. Louis, MO, USA), and 18.9 g/L d-glucose (Sigma-Aldrich, F0127, St. Louis, MO, USA)) for 7 months.

### Western blot analysis

Western blot analyses were performed as described in a previous study [53]. The protein concentrations of cell or tissue lysates were measured using the Lowry assay. Depending on the molecular weight of the target protein, 10 μg protein samples were separated using 8% or 12% sodium dodecyl sulfate polyacrylamide gel electrophoresis (SDS PAGE) and electroblotted onto a nitrocellulose membrane. The membranes were blocked with 5% non-fat dry milk in Tris buffered saline plus Tween (TBST), immunoblotted with specific primary antibodies against CAV-1 (GTX79350, GeneTex, Irvine, CA, USA), eNOS (A15075, ABclonal, Woburn, MA, USA), BNP (1:500, sc-18813, Santa Cruz Biotechnology, Santa Cruz, CA, USA), cytochrome c (1:1000, sc-13156, Santa Cruz), caspase 3 (1:1000, sc-56053, Santa Cruz), and β-actin (1:1000, T5168, GeneTex), and detected using horseradish peroxidase conjugated secondary antibodies. Fluorography with an enhanced detection kit was used to visualize the signals (ECL, GE Healthcare Life Sciences, Buckinghamshire Amersham Pharmacia International).

### Transmission electron microscopy (TEM) assay

TEM was performed as described in a previous study [53]. Briefly, tissue samples were fixed with 2.5% glutaraldehyde for 2 h at 4 °C. After washing, the samples were post-fixed in 1% osmium tetroxide for 2 h, dehydrated in graded acetone, infiltrated, and then embedded in Epoxy resin. Ultrathin 70 nm sections were cut using a Leica microtome (Leica RM2165, Tokyo, Japan) and examined using an FE TEM (HITACHI HT 7700, Tokyo, Japan) at an accelerating voltage of 80 kV.

### Histology, immunohistochemistry, and Oil Red O staining

Parts of the heart were fixed with 4% paraformaldehyde and then wax embedded and sectioned (5 μm thick) for hematoxylin and eosin staining (H&E, Invitrogen, Carlsbad, CA, USA), as previously described [54]. The lipid content was quantified by Oil Red O staining (Bio Vision, Catalog # K58024, Mountain View, CA, USA). After staining with hematoxylin and washing with dH_2_O, the section samples were washed and treated with 60% isopropanol for 5 min each time with gentle rocking. Thereafter, the section samples were extracted after Oil Red O staining with 100% isopropanol for 5 min with gentle rocking. Immunohistochemical staining was used to measure autophagic or apoptotic protein expression, with tissue sections (5 μm thick) incubated in blocking buffer (0.5% BSA, 0.05% Tween 20, and PBS) for 1 h at room temperature, followed by specific primary antibodies against BNP (1:100, sc-271185, Santa Cruz), cytochrome c (1:100, sc-13156, Santa Cruz), and caspase 3 (1:100, sc-56053, Santa Cruz) for 1 h at room temperature. The antibody staining was developed using a diaminobenzidine (DAB) detection system (Catalog # 760124, Ventana Medical Systems, Tucson, AZ, USA) according to the manufacturer's protocol, counterstained with hematoxylin, and examined under a microscope (Nikon E600, Japan).

### Immunostaining

Immunostaining was performed as described in a previous study [53]. First, paraffin tissue sections (5 µm-thick) were incubated in a blocking buffer for 1 h, at room temperature. Second, sections or cells were incubated for 1 h with primary antibodies against CAV-1 (1:100, GTX79350, GeneTex), VE-cadherin (1:100, GTX633705, GeneTex), β-catenin (1:100, sc-7199, Santa Cruz Biotechnology), mitochondrial antiviral-signaling protein (MAVS) (1:100, Cat #PA5-17256, Invitrogen), Mitofusin 2 (MFN2) (1:100, sc-100560, Santa Cruz), and lipid (70065-T, Biotium, Fremont, CA, USA). Finally, the cell nuclei were counterstained with 4ʹ,6-diamidino-2-phenylindole, washed, mounted with VECTASHIELD^®^ mounting medium (Vector Laboratories, Burlingame, CA, USA), and examined under a fluorescence microscope (Leica, Wetzlar, Germany). Confocal images were analyzed using the Imaris 3D/4D analysis software (OXFORD instruments, USA).

### Data and statistical analysis

Numerical values are reported as mean ± standard deviation (SD) or mean ± standard error of the mean (SEM). Statistical analyses were performed using the GraphPad Prism 7.0. Unless otherwise stated in the figure legends, statistical significance (*P < 0.05; **P < 0.01; ***P < 0.001, ****P < 0.001) was determined using unpaired two-tailed *t*-test or one-way ANOVA with relevant post-hoc tests (Dunnett, unless specified otherwise).

## Data Availability

Not applicable.

## Abbreviations

WD: Western diet
MS: Metabolic syndrome
LD: Lipid droplet
L: Lysosome
GOT: Glutamic oxaloacetic transaminase
TG: Triglycerides
GPT: Glutamic pyruvic transaminase
VVO: Vesiculo-vacuolar organelle
CAV-1: Caveolin-1
VLDL: Very-low-density lipoprotein
LDL: Low-density lipoprotein
TEM: Transmission electron microscope
eNOS: Endothelial nitric oxide synthase
NO: Nitric oxide
EC: Endothelial cells
ECM: Extracellular matrix
MAM: Mitochondria-associated endoplasmic reticulum membrane
MST: Mitochondrial shape transition
ER: Endoplasmic reticulum
M: Mitochondria
V: Vascular
CM: Cardiomyocyte
MAVS: Mitochondrial antiviral signaling
MFN2: Mitofusin 2
DAPI: 4′,6-Diamidino-2-phenylindole
LVESd: Left ventricular end-systolic diameter
IVSd: Inter-ventricular septal thickness in diastole
IVSs: Inter-ventricular septal thickness in systole
PWTd: Posterior LV wall thickness in diastole
PWTs: Posterior LV wall thickness in systole
BNP: B-type natriuretic peptide
Cyt *c*: Cytochrome *c*
Casp-3: Caspase-3
ROS: Reactive oxygen species

## References

[1] de Souza GM, de Albuquerque Borborema ME, de Lucena TMC, da Silva Santos AF, de Lima BR, de Oliveira DC, de Azevêdo SJ (2020). Caveolin-1 (CAV-1) up regulation in metabolic syndrome: all roads leading to the same end. Mol Biol Rep.

[2] Yang Y, Kurian J, Schena G, Johnson J, Kubo H, Travers JG, Kang C, Lucchese AM, Eaton DM, Lv M (2021). Cardiac remodeling during pregnancy with metabolic syndrome: prologue of pathological remodeling. Circulation.

[3] Mouton AJ, Li X, Hall ME, Hall JE (2020). Obesity, hypertension, and cardiac dysfunction: novel roles of immunometabolism in macrophage activation and inflammation. Circ Res.

[4] Russell J, Du Toit EF, Peart JN, Patel HH, Headrick JP (2017). Myocyte membrane and microdomain modifications in diabetes: determinants of ischemic tolerance and cardioprotection. Cardiovasc Diabetol.

[5] Satoh T, Wang L, Espinosa-Diez C, Wang B, Hahn SA, Noda K, Rochon ER, Dent MR, Levine AR, Baust JJ (2021). Metabolic syndrome mediates ROS-miR-193b-NFYA-dependent downregulation of soluble guanylate cyclase and contributes to exercise-induced pulmonary hypertension in heart failure with preserved ejection fraction. Circulation.

[6] Zhang X, Liu H, Hao Y, Xu L, Zhang T, Liu Y, Guo L, Zhu L, Pei Z (2018). Coenzyme Q10 protects against hyperlipidemia-induced cardiac damage in apolipoprotein E-deficient mice. Lipids Health Dis.

[7] Taskaeva I, Bgatova N (2021). Microvasculature in hepatocellular carcinoma: an ultrastructural study. Microvasc Res.

[8] LeVine DN, Cianciolo RE, Linder KE, Bizikova P, Birkenheuer AJ, Brooks MB, Salous AK, Nordone SK, Bellinger DA, Marr H (2019). Endothelial alterations in a canine model of immune thrombocytopenia. Platelets.

[9] Gumbleton M, Abulrob AG, Campbell L (2000). Caveolae: an alternative membrane transport compartment. Pharm Res.

[10] Parton RG, Tillu VA, Collins BM (2018). Caveolae. Curr Biol CB.

[11] Harding IC, Mitra R, Mensah SA, Herman IM, Ebong EE (2018). Pro-atherosclerotic disturbed flow disrupts caveolin-1 expression, localization, and function via glycocalyx degradation. J Transl Med.

[12] Yokomori H, Ando W, Oda M (2019). Caveolin-1 is related to lipid droplet formation in hepatic stellate cells in human liver. Acta Histochem.

[13] Raudenska M, Gumulec J, Balvan J, Masarik M (2020). Caveolin-1 in oncogenic metabolic symbiosis. Int J Cancer.

[14] Nwosu ZC, Ebert MP, Dooley S, Meyer C (2016). Caveolin-1 in the regulation of cell metabolism: a cancer perspective. Mol Cancer.

[15] Núñez-Wehinger S, Ortiz RJ, Díaz N, Díaz J, Lobos-González L, Quest AF (2014). Caveolin-1 in cell migration and metastasis. Curr Mol Med.

[16] Cyr AR, Huckaby LV, Shiva SS, Zuckerbraun BS (2020). Nitric oxide and endothelial dysfunction. Crit Care Clin.

[17] Xu S, Ilyas I, Little PJ, Li H, Kamato D, Zheng X, Luo S, Li Z, Liu P, Han J (2021). Endothelial dysfunction in atherosclerotic cardiovascular diseases and beyond: from mechanism to pharmacotherapies. Pharmacol Rev.

[18] Jia G, Aroor AR, Jia C, Sowers JR (2019). Endothelial cell senescence in aging-related vascular dysfunction. Biochim Biophys Acta Mol Basis Dis.

[19] Grandl G, Wolfrum C (2018). Hemostasis, endothelial stress, inflammation, and the metabolic syndrome. Semin Immunopathol.

[20] Horton WB, Barrett EJ (2021). Microvascular dysfunction in diabetes mellitus and cardiometabolic disease. Endocr Rev.

[21] Fenton AR, Jongens TA, Holzbaur ELF (2021). Mitochondrial dynamics: shaping and remodeling an organelle network. Curr Opin Cell Biol.

[22] Yang M, Li C, Sun L (2021). Mitochondria-associated membranes (MAMs): a novel therapeutic target for treating metabolic syndrome. Curr Med Chem.

[23] Gao P, Yan Z, Zhu Z (2020). Mitochondria-associated endoplasmic reticulum membranes in cardiovascular diseases. Front Cell Dev Biol.

[24] Silva-Palacios A, Zazueta C, Pedraza-Chaverri J (2020). ER membranes associated with mitochondria: possible therapeutic targets in heart-associated diseases. Pharmacol Res.

[25] Ait-Aissa K, Nguyen QM, Gabani M, Kassan A, Kumar S, Choi SK, Gonzalez AA, Khataei T, Sahyoun AM, Chen C (2020). MicroRNAs and obesity-induced endothelial dysfunction: key paradigms in molecular therapy. Cardiovasc Diabetol.

[26] Incalza MA, D'Oria R, Natalicchio A, Perrini S, Laviola L, Giorgino F (2018). Oxidative stress and reactive oxygen species in endothelial dysfunction associated with cardiovascular and metabolic diseases. Vascul Pharmacol.

[27] Shamsaldeen YA, Lione LA, Benham CD (2020). Dysregulation of TRPV4, eNOS and caveolin-1 contribute to endothelial dysfunction in the streptozotocin rat model of diabetes. Eur J Pharmacol.

[28] Shamsaldeen YA, Ugur R, Benham CD, Lione LA (2018). Diabetic dyslipidaemia is associated with alterations in eNOS, caveolin-1, and endothelial dysfunction in streptozotocin treated rats. Diabetes Metab Res Rev.

[29] Zhang X, Fernández-Hernando C (2020). Transport of LDLs into the arterial wall: impact in atherosclerosis. Curr Opin Lipidol.

[30] Krols M, van Isterdael G, Asselbergh B, Kremer A, Lippens S, Timmerman V, Janssens S (2016). Mitochondria-associated membranes as hubs for neurodegeneration. Acta Neuropathol.

[31] White CR, Datta G, Giordano S (2017). High-density lipoprotein regulation of mitochondrial function. Adv Exp Med Biol.

[32] Youle RJ, van der Bliek AM (2012). Mitochondrial fission, fusion, and stress. Science.

[33] Montaigne D, Marechal X, Coisne A, Debry N, Modine T, Fayad G, Potelle C, El Arid JM, Mouton S, Sebti Y (2014). Myocardial contractile dysfunction is associated with impaired mitochondrial function and dynamics in type 2 diabetic but not in obese patients. Circulation.

[34] Engin AB (2017). What is lipotoxicity?. Adv Exp Med Biol.

[35] Nishi H, Higashihara T, Inagi R (2019). Lipotoxicity in kidney, heart, and skeletal muscle dysfunction. Nutrients.

[36] Magnifico MC, Oberkersch RE, Mollo A, Giambelli L, Grooten Y, Sarti P, Calabrese GC, Arese M (2017). VLDL induced modulation of nitric oxide signalling and cell redox homeostasis in HUVEC. Oxid Med Cell Longev.

[37] Tenenbaum A, Klempfner R, Fisman EZ (2014). Hypertriglyceridemia: a too long unfairly neglected major cardiovascular risk factor. Cardiovasc Diabetol.

[38] Bhanpuri NH, Hallberg SJ, Williams PT, McKenzie AL, Ballard KD, Campbell WW, McCarter JP, Phinney SD, Volek JS (2018). Cardiovascular disease risk factor responses to a type 2 diabetes care model including nutritional ketosis induced by sustained carbohydrate restriction at 1 year: an open label, non-randomized, controlled study. Cardiovasc Diabetol.

[39] Mathew R (2021). Critical role of caveolin-1 loss/dysfunction in pulmonary hypertension. Med Sci.

[40] Rutkowski JM, Halberg N, Wang QA, Holland WL, Xia JY, Scherer PE (2014). Differential transendothelial transport of adiponectin complexes. Cardiovasc Diabetol.

[41] Zhang X, Ramirez CM, Aryal B, Madrigal-Matute J, Liu X, Diaz A, Torrecilla-Parra M, Suarez Y, Cuervo AM, Sessa WC (2020). Cav-1 (Caveolin-1) deficiency increases autophagy in the endothelium and attenuates vascular inflammation and atherosclerosis. Arterioscler Thromb Vasc Biol.

[42] Wang S, Ichinomiya T, Terada Y, Wang D, Patel HH, Head BP (2021). Synapsin-promoted caveolin-1 overexpression maintains mitochondrial morphology and function in PSAPP Alzheimer's disease mice. Cells.

[43] Yu DM, Jung SH, An HT, Lee S, Hong J, Park JS, Lee H, Lee H, Bahn MS, Lee HC (2017). Caveolin-1 deficiency induces premature senescence with mitochondrial dysfunction. Aging Cell.

[44] Ilha M, Meira Martins LA, da Silveira MK, Dias CK, Thomé MP, Petry F, Rohden F, Borojevic R, Trindade VMT, Klamt F (2022). Caveolin-1 influences mitochondrial plasticity and function in hepatic stellate cell activation. Cell Biol Int.

[45] Zeng W, Tang J, Li H, Xu H, Lu H, Peng H, Lin C, Gao R, Lin S, Lin K (2018). Caveolin-1 deficiency protects pancreatic β cells against palmitate-induced dysfunction and apoptosis. Cell Signal.

[46] Fernández Casafuz AB, De Rossi MC, Bruno L (2021). Morphological fluctuations of individual mitochondria in living cells. J Phys Condens Matter.

[47] Nemani N, Carvalho E, Tomar D, Dong Z, Ketschek A, Breves SL, Jaña F, Worth AM, Heffler J, Palaniappan P (2018). MIRO-1 determines mitochondrial shape transition upon GPCR activation and Ca(2+) stress. Cell Rep.

[48] Sun X, Alford J, Qiu H (2021). Structural and functional remodeling of mitochondria in cardiac diseases. Int J Mol Sci.

[49] Kaludercic N, Di Lisa F (2020). Mitochondrial ROS formation in the pathogenesis of diabetic cardiomyopathy. Front Cardiovasc Med.

[50] Brandt T, Mourier A, Tain LS, Partridge L, Larsson NG, Kühlbrandt W (2017). Changes of mitochondrial ultrastructure and function during ageing in mice and drosophila. Elife.

[51] Pfluger PT, Kabra DG, Aichler M, Schriever SC, Pfuhlmann K, García VC, Lehti M, Weber J, Kutschke M, Rozman J (2015). Calcineurin links mitochondrial elongation with energy metabolism. Cell Metab.

[52] Tsuchida T, Lee YA, Fujiwara N, Ybanez M, Allen B, Martins S, Fiel MI, Goossens N, Chou HI, Hoshida Y (2018). A simple diet- and chemical-induced murine NASH model with rapid progression of steatohepatitis, fibrosis and liver cancer. J Hepatol.

[53] Hsieh CC, Li CY, Hsu CH, Chen HL, Chen YH, Liu YP, Liu YR, Kuo HF, Liu PL (2019). Mitochondrial protection by simvastatin against angiotensin II-mediated heart failure. Br J Pharmacol.

[54] Kuo HF, Hsieh CC, Wang SC, Chang CY, Hung CH, Kuo PL, Liu YR, Li CY, Liu PL (2019). Simvastatin attenuates cardiac fibrosis via regulation of cardiomyocyte-derived exosome secretion. J Clin Med.

